# Fiber-Optic Sensor-Based Structural Health Monitoring with Machine Learning: A Task-Oriented and Cross-Domain Review

**DOI:** 10.3390/s26092641

**Published:** 2026-04-24

**Authors:** Yasir Mahmood, Nof Yasir, Kathryn Quenette, Gul Badin, Ying Huang, Luyang Xu

**Affiliations:** 1 Department of Civil, Construction, and Environmental Engineering, North Dakota State University, Fargo, ND 58102, USA; yasir.mahmood@ndsu.edu (Y.M.); kathryn.quenette@ndsu.edu (K.Q.); gul.badin@ndsu.edu (G.B.); ying.huang@ndsu.edu (Y.H.); 2 Department of Electrical and Computer Engineering, North Dakota State University, Fargo, ND 58102, USA; nof.yasir@ndsu.edu

**Keywords:** distributed fiber optic sensing, fiber Bragg grating, damage detection, data-driven modeling, digital twin, physics-informed learning, infrastructure resilience, spatiotemporal monitoring

## Abstract

**Highlights:**

- Link fiber-optic sensing configurations with machine-learning paradigms in SHM.
- Map FOS data structures to SHM tasks (detection, localization, prognosis).
- Integrate sensing physics and ML across major infrastructure domains.
- Discuss deployment challenges in data scale, environmental variability, and model transferability.
- Identify future directions, including physics-informed learning and digital twins.

**Simple Summary:**

Structural health monitoring (SHM) is critical to the safety and longevity of modern infrastructure, including bridges, pipelines, and transportation networks. Fiber-optic sensors (FOSs) have emerged as a powerful technology for SHM, providing accurate, real-time measurements even in harsh environments. At the same time, ML techniques have significantly improved the analysis of large, complex datasets generated by these sensors. This review paper provides a comprehensive overview of integrating fiber-optic sensing technologies with machine learning for intelligent SHM applications. It summarizes recent advances, highlights key challenges, and discusses future research directions across multiple infrastructure domains. The findings aim to support the development of more reliable, data-driven monitoring systems for resilient infrastructure.

**Abstract:**

Structural health monitoring (SHM) plays an increasingly important role in managing aging, safety-critical infrastructure under growing environmental and operational demands. In recent years, fiber-optic sensors (FOSs) have attracted significant attention for SHM applications due to their immunity to electromagnetic interference, durability in harsh environments, multiplexing capability, and suitability for both localized and fully distributed measurements. In parallel, advances in machine learning (ML) have enabled new approaches for extracting actionable insights from large, high-dimensional sensing datasets. This paper presents a systematic review of FOS-based SHM systems integrated with ML across civil, transportation, energy, marine, and aerospace infrastructures. Following PRISMA 2020 guidelines, peer-reviewed studies were identified and synthesized to examine sensing principles, deployment configurations, data characteristics, and learning-based analytical strategies. Fiber optic technologies are categorized into point-based, quasi-distributed, and fully distributed systems, and their capabilities for capturing strain, temperature, and spatiotemporal structural responses are critically evaluated. ML approaches are examined from a task-oriented perspective, including damage detection, localization, severity assessment, environmental compensation, and prognosis, with emphasis on the alignment between sensing configurations and appropriate learning paradigms. Key challenges remain, particularly regarding large data volumes, environmental variability, limited labeled damage datasets, model generalization, and system-level integration. Emerging directions such as physics-informed and hybrid learning, transfer learning, uncertainty-aware modeling, and integration with digital twins are discussed as pathways toward more robust and scalable SHM systems. By jointly addressing sensing physics and data-driven intelligence, this review provides a structured reference and practical roadmap for advancing intelligent FOS-based SHM in next-generation infrastructure.

## 1. Introduction

Civil, transportation, energy, marine, and aerospace infrastructures form the backbone of modern society. Many of these systems, however, are now operating under conditions that differ substantially from those assumed at the time of their design [1,2]. Aging materials, increased traffic demands, harsher environmental exposure, and more frequent extreme weather events have placed additional stress on structural components [3,4,5]. As a result, the likelihood of deterioration and unexpected failure has become a growing concern. When such failures occur, the consequences may include safety risks, service disruption, environmental damage, and significant economic loss. These realities underscore the need for monitoring approaches that support timely intervention and informed lifecycle management decisions [6,7].

Structural Health Monitoring (SHM) has emerged as a practical framework to address this need by enabling continuous assessment of structural condition and performance [8,9]. Rather than relying solely on periodic inspections, modern SHM approaches increasingly integrate sensing technologies, data acquisition systems, and analytical models to enable more continuous assessment [1,3,10]. The primary goals of SHM include early damage detection, performance evaluation, and maintenance planning, all of which contribute to improved safety and resource allocation.

**Figure 1:**
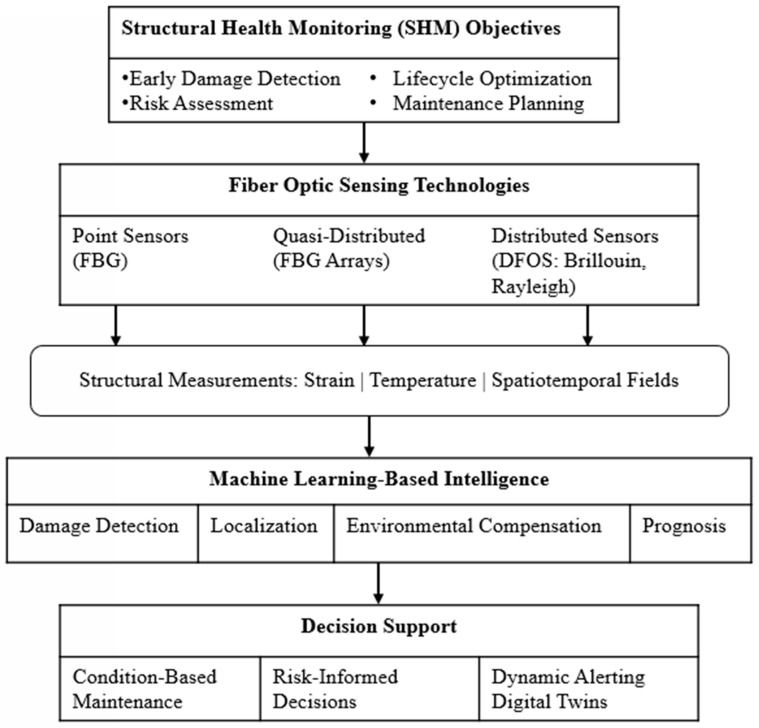
Conceptual overview of fiber optic sensor-based SHM integrated with machine learning.

Traditional SHM implementations have relied largely on electrical strain gauges, accelerometers, piezoelectric transducers, acoustic emission sensors, and visual inspection techniques [11,12,13]. These tools have played a foundational role in advancing the field. However, their application to large-scale or long-term monitoring can be constrained by wiring complexity, susceptibility to electromagnetic interference, durability limitations in harsh environments, and relatively sparse spatial coverage [14,15,16,17]. Such limitations are particularly evident in distributed or safety-critical infrastructure, motivating the exploration of alternative sensing technologies. To address these challenges, fiber-optic sensor (FOS) technologies have gained attention as key enabling technologies in modern SHM systems [18]. Figure 1 illustrates the conceptual framework of FOS-based SHM integrated with ML. The first tier shows the SHM objective, which mainly includes damage detection, risk assessment, lifecycle optimization, and maintenance planning. The second tier shows three principal categories of fiber-optic sensing technologies: point sensors such as fiber Bragg gratings (FBGs), quasi-distributed sensors (e.g., FBG arrays), and distributed fiber-optic sensing (DFOS) systems based on Rayleigh, Brillouin, or Raman scattering [19,20,21].

As shown in Figure 1, fiber-optic sensing technologies serve as the interface between SHM objectives and structural measurements, generating rich spatiotemporal datasets across a wide range of infrastructure domains, including bridges, buildings, tunnels, dams, pavements, railways, offshore platforms, subsea pipelines, wind turbines, and aerospace structures. While such data provide unprecedented insight into structural behavior, they also introduce significant challenges in interpretation rather than in data acquisition [22,23,24]. One challenge is that FOS-based SHM systems employing distributed sensing generate large volumes of high-dimensional data with strong spatial and temporal correlations [25,26]. Distinguishing damage-related signatures from environmental and operational variability, managing nonlinearity and noise, and translating raw measurements into actionable information remain major obstacles [27,28]. Conventional signal processing and threshold-based approaches often struggle under real-world operating conditions [29,30]. To address these analytical challenges, ML has become increasingly integrated into SHM systems [31,32], as reflected in the fourth tier of Figure 1. Studies over the past decade show that data-driven and hybrid learning approaches can support tasks such as damage detection, localization, environmental compensation, and structural prognosis when applied to fiber-optic measurements [14,15,33,34,35]. Techniques including artificial neural networks (ANNs), support vector machines (SVMs), convolutional models, and physics-informed frameworks allow complex patterns to be identified without relying solely on predefined analytical thresholds [19,35,36,37].

The integration of SHM objectives, fiber optic sensing, and ML-based intelligence ultimately aims to enable decision support, as shown in the bottom tier of Figure 1. These decision-oriented outcomes, including condition-based maintenance, risk-informed decision-making, dynamic alerting, and digital twin integration, represent the practical realization of intelligent SHM systems and are increasingly emphasized as essential for improving infrastructure resilience and long-term asset management [38,39,40,41].

Although research in SHM, fiber-optic sensing, and machine learning has advanced considerably, most existing reviews still treat these areas separately rather than examining their integration. A comprehensive synthesis that explicitly connects SHM objectives, sensing modalities, structural measurements, machine-learning interpretation, and decision-support outcomes across multiple infrastructure domains remains limited.

To address this gap, the present review adopts a task-oriented perspective that maps fiber-optic data types from point-based FBG measurements to distributed spatiotemporal fields and aligns them with ML paradigms with core SHM objectives. It further provides a cross-infrastructure synthesis spanning civil, transportation, energy, marine, and aerospace systems, with attention to deployment readiness and practical implementation challenges. Finally, the review integrates sensing physics, data-driven analytics, and decision-support considerations into a unified framework. It outlines emerging directions, such as physics-informed learning and digital twin-enabled SHM.

This review examines how fiber-optic sensing technologies and ML methods are being combined for SHM of modern infrastructure systems. Unlike many existing reviews that focus primarily on sensing technologies or data-analysis methods individually, this paper adopts a task-oriented perspective that links sensing configurations, data characteristics, and ML approaches within SHM workflows. By synthesizing studies across multiple infrastructure domains, the review aims to clarify current research trends, identify practical challenges in field deployment, and highlight promising directions for intelligent SHM systems. To provide a structured overview of this rapidly evolving field, this review adopts a systematic literature screening approach supported by bibliometric mapping and thematic analysis.

This review does not aim to provide an exhaustive treatment of each individual domain (e.g., fiber-optic sensing physics or machine learning theory) in isolation. Instead, it focuses on integrating these components through a task-oriented perspective, highlighting how sensing configurations, data characteristics, and learning methodologies interact to enable effective structural health monitoring across different infrastructure systems.

## 2. Review Methodology

### 2.1. Literature Search Strategy

This review was conducted in accordance with the Preferred Reporting Items for Systematic Reviews and Meta-Analyses (PRISMA 2020) guidelines to ensure transparency and reproducibility in the literature identification and screening process. The systematic search focused on studies that integrated fiber-optic sensing technologies with machine learning methods for structural health monitoring applications.

To ensure a focused yet comprehensive review, the literature search was organized around three main themes: (1) fiber-optic sensing technologies, (2) structural health monitoring applications, and (3) machine-learning or data-driven analysis methods. For each theme, closely related terms and synonyms were grouped together using the Boolean operator “OR,” while the three themes were connected using “AND” to retrieve studies located at their intersection.

The search was conducted in the Web of Science (WoS) database using the Topic (TS) field, which includes titles, abstracts, author keywords, and Keywords Plus. Table 1 lists the keyword categories and representative search terms used in the query formulation. To avoid retrieving an overly broad set of unrelated machine-learning studies, generic expressions such as “damage detection” were not included in the initial search string. Instead, relevance was evaluated during the screening stage to confirm that selected studies explicitly involved fiber-optic sensing within an SHM context. This review was not registered in a systematic review registry (e.g., PROSPERO) because it focuses on engineering and infrastructure monitoring studies rather than clinical research.

The WoS Core Collection was selected as the primary database due to its comprehensive coverage of high-quality, peer-reviewed literature across engineering, materials science, and interdisciplinary domains. While using a single database may limit inclusion of some studies indexed elsewhere, this approach ensures consistency, reproducibility, and a focus on well-established sources within the field.

### 2.2. Study Selection and Screening Process

The literature selection and screening process followed the PRISMA 2020 framework to ensure transparency and reproducibility in the identification and evaluation of studies. Studies were included if they addressed (i) structural health monitoring applications, (ii) fiber-optic sensing technologies, and (iii) machine-learning-based data analysis methods. Articles not related to SHM, lacking fiber-optic sensing components, or not involving machine-learning techniques were excluded during the screening process. Only peer-reviewed journal articles written in English were considered to ensure consistency and quality of the analyzed literature.

The initial search of the Web of Science Core Collection returned 3096 records using the predefined search strategy. After removing 412 duplicate records, a total of 2684 records remained for title and abstract screening. During this stage, 2360 records were excluded because they were not directly related to fiber-optic sensing technologies, structural health monitoring applications, or machine-learning-based analysis methods.

The remaining 324 articles were considered potentially relevant and were retrieved for full-text evaluation. Among these, 18 records could not be retrieved, leaving 306 articles for full-text eligibility assessment. After detailed evaluation, 188 articles were excluded due to the absence of fiber-optic sensing components, lack of an SHM context, absence of machine-learning methods, non-peer-reviewed publication types, or insufficient methodological detail. Finally, 118 studies met the inclusion criteria and were incorporated into the qualitative synthesis of this review.

**Figure 2:**
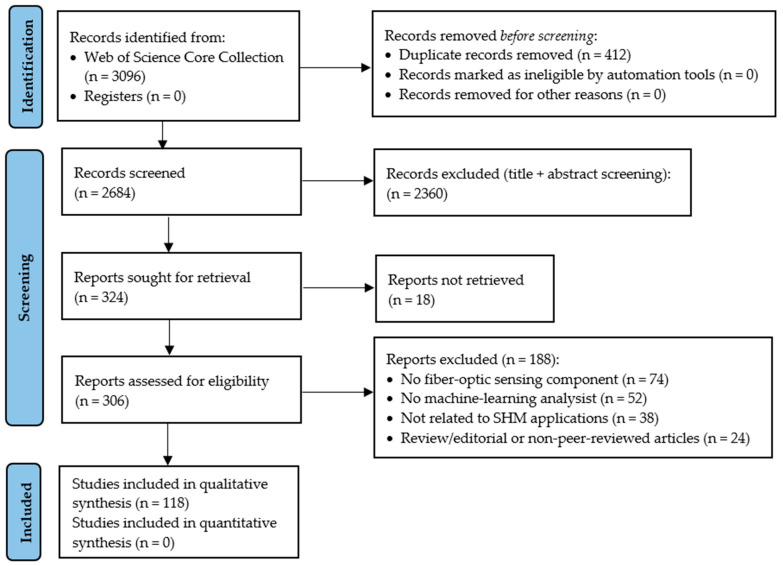
PRISMA 2020 flow diagram illustrating the identification, screening, eligibility assessment, and final inclusion of studies used for the qualitative synthesis in this review.

These 118 studies represent the core dataset used for the systematic synthesis of fiber-optic sensor-based structural health monitoring combined with machine learning. Additional references cited throughout the manuscript provide supporting background on SHM principles, fiber-optic sensing technologies, machine-learning methods, and domain-specific infrastructure applications. The complete identification, screening, and eligibility assessment process is illustrated in the PRISMA flow diagram (Figure 2).

In addition to relevance screening, the included studies were qualitatively evaluated to ensure they provided sufficient methodological detail on fiber-optic sensing technologies, SHM applications, and machine-learning-based analysis.

In addition to relevance screening, the included studies were qualitatively evaluated to ensure they provided sufficient methodological detail on fiber-optic sensing technologies, SHM applications, and machine-learning-based analysis. The final set of included studies formed the basis for the qualitative synthesis and thematic analysis presented in the subsequent sections of this review. The PRISMA 2020 checklist is provided in the Supplementary Materials (File S1).

It is important to note that the literature selection was based on a single database and predefined search criteria, which may not capture all relevant studies. However, the adopted approach provides a balanced and reproducible dataset for qualitative synthesis. Future work may expand the search across multiple databases and incorporate quantitative quality assessment metrics to further enhance methodological rigor.

### 2.3. Bibliometric Mapping and Keyword Analysis

In addition to the qualitative synthesis, bibliometric analysis was performed using the WoS database to better understand how research at the intersection of SHM, fiber-optic sensing, and ML has evolved. Publication trends from 2005 to 2025 were examined, together with cumulative outputs from different Boolean search combinations. Science-mapping tools in VOSviewer 1.6.20. were used to explore keyword co-occurrence patterns and country-level collaboration networks.

**Figure 3:**
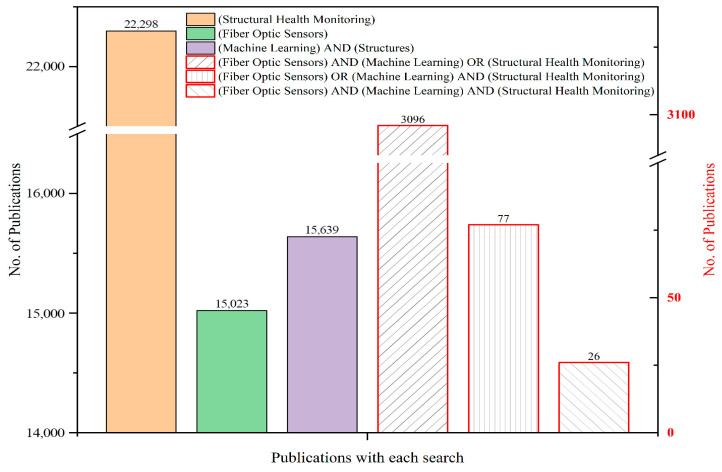
Total WoS publications (2005–2025) for six SHM/FOS/ML search strands.

The broader parent domains SHM, fiber-optic sensing, and ML applied to structures have all grown steadily over the past two decades, as shown in Figure 3. However, when the three themes are combined using the strict query “(Fiber-optic sensors) AND (Machine Learning) AND (Structural Health Monitoring),” the number of publications remains comparatively small. While there has been a slight increase in recent years, the overall count remains limited compared to the individual domains.

For example, over the full 2005–2025 period, “Structural Health Monitoring” alone yields 22,298 records, “Machine Learning” AND “Structures” yields 15,639, and “Fiber-optic sensors” yields 15,023. The strict triple-intersection produces only a few dozen records over two decades, underscoring how specialized this niche remains. Even broader combined queries, such as “(Fiber-optic sensors) AND (Machine Learning) OR (Structural Health Monitoring),” return 3096 records, while more restrictive combinations drop sharply to fewer than 100. These differences highlight how relatively small the explicitly integrated SHM–FOS–ML literature remains when compared with the much larger parent fields.

It is important to note that WoS Topic searches depend on the explicit appearance of terms in titles, abstracts, and keywords. As a result, some relevant studies may not be captured if they use alternative terminology (for example, specific FOS modalities or different SHM phrasing). The trends shown in Figure 3, therefore, reflect how often the three themes are explicitly labeled together rather than the absolute extent of technical overlap.

**Figure 4:**
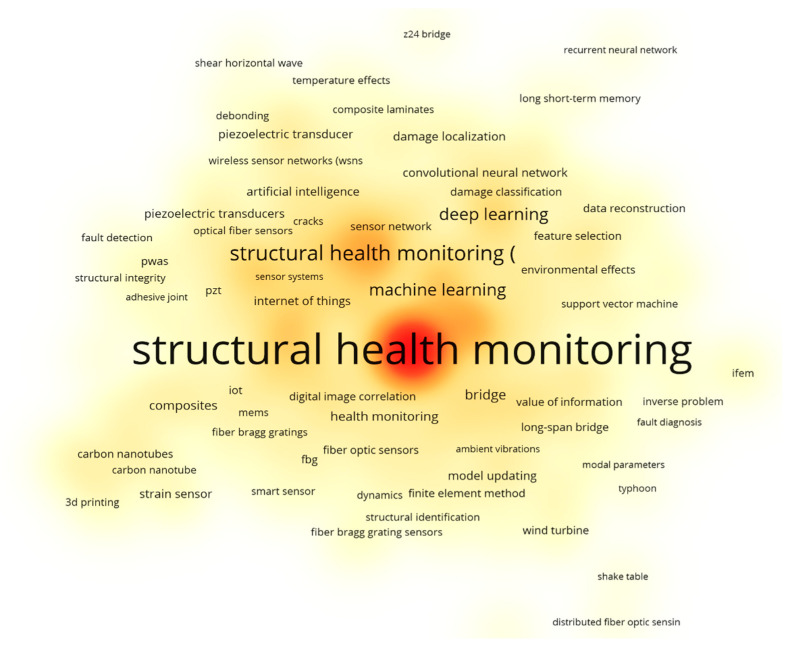
Density map of author-keyword co-occurrence for fiber-optic-sensor and machine-learning-enabled SHM research (minimum occurrence = 5).

Figure 4 presents a density visualization of author-keyword co-occurrence generated in VOSviewer, with a minimum occurrence threshold of 5. “Structural Health Monitoring” appears as the most central and densely connected term, serving as the primary anchor of the dataset. Closely associated terms include “machine learning” and “deep learning,” indicating that data-driven methods are now frequently discussed in SHM research.

Around this central cluster, algorithm-specific keywords such as convolutional neural networks (CNNs), long short-term memory (LSTM), and SVMs appear alongside application-oriented terms such as damage detection, damage localization, and feature selection. This pattern reflects the emphasis on extracting diagnostic information from monitoring data. Fiber-optic terms such as fiber Bragg grating and distributed fiber-optic sensing are clustered together, indicating that FOS technologies are commonly studied alongside learning-based approaches.

In addition, structural and mechanics-related keywords (e.g., bridge, composite laminates, cracks, debonding, finite element method, and model updating) remain present. This indicates that many contributions continue to link data-driven analysis with underlying structural behavior and modeling, rather than treating ML as a standalone analytical tool.

**Figure 5:**
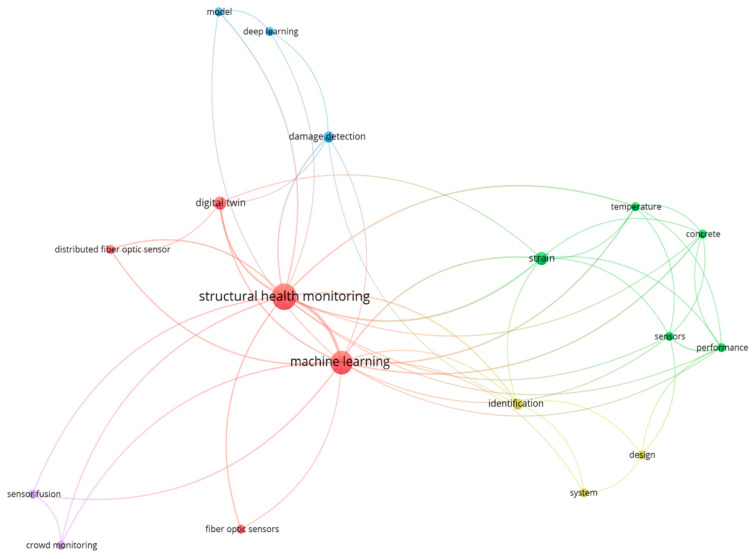
Author-keyword co-occurrence network for the strict SHM–ML–FOS intersection set (N = 26; minimum occurrence = 2).

Figure 5 narrows the analysis to publications retrieved using the strict triple-intersection query. Because this subset contains only 26 records over the 2005–2025 period, the minimum keyword-occurrence threshold was reduced to 2. The resulting network should therefore be interpreted as illustrative rather than exhaustive.

Within this focused map, “Structural Health Monitoring” remains the central node and is directly connected to “machine learning,” reinforcing that studies at this intersection are typically framed as SHM problems rather than purely sensing or algorithmic investigations. Terms such as “deep learning” and “damage detection” appear prominently, reflecting the dominant focus on diagnostic tasks.

On the sensing side, keywords including “distributed fiber optic sensor,” “sensor fusion,” and “digital twin” suggest growing interest in integrating fiber-optic measurements into broader system architectures. At the same time, frequently recurring measurement-related terms such as strain, temperature, and concrete indicate that much of the current work remains focused on traditional civil engineering applications.

**Figure 6:**
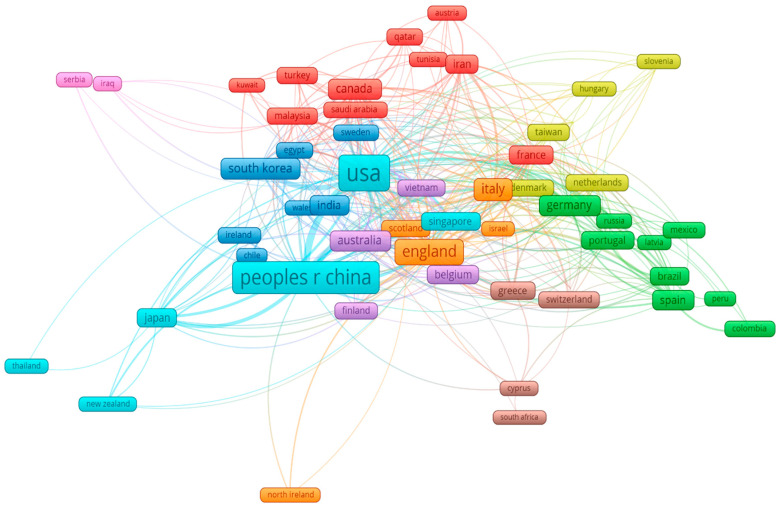
Country-level productivity, citations, and collaboration network in the retrieved dataset.

Figure 6 summarizes country-level productivity and collaboration patterns within the retrieved dataset. The United States and the People’s Republic of China account for the largest publication outputs (756 and 781 documents, respectively) and the highest citation counts (35,402 and 23,849). England and Italy follow as the next most active contributors, with Germany and Australia also showing substantial activity.

Beyond publication volume, collaboration link strength in the VOSviewer network highlights the interconnected nature of the research community. The United States (total link strength: 389) and China (307) appear as central collaboration hubs, followed by England (196) and Italy (123). This pattern indicates that research in this area is supported by a relatively concentrated set of leading countries that maintain active international partnerships.

Overall, the bibliometric evidence suggests that while SHM, fiber-optic sensing, and machine learning are each well-established research domains, their explicit three-way integration remains comparatively limited. At the same time, recent increases in co-occurrence and collaboration point toward the gradual consolidation of this intersection as a recognizable research direction.

Following the screening and bibliometric assessment stages, the selected studies were analyzed thematically to identify key developments in fiber-optic sensing technologies, ML-based SHM approaches, and cross-domain infrastructure applications. The findings of this analysis are organized and discussed in the subsequent sections of the paper. The selected studies were subsequently analyzed to identify key developments in fiber-optic sensing technologies, machine-learning-based SHM methods, and cross-domain infrastructure applications.

Rather than applying formal quality scoring metrics, the included studies were qualitatively evaluated based on their relevance to fiber-optic sensing configurations, structural health monitoring objectives, and machine learning methodologies. This approach enables a structured comparison aligned with the task-oriented framework adopted in this review.

## 3. SHM: Background, Concepts, and Sensing Systems

### 3.1. Background and Evolution of SHM

SHM has emerged as a multidisciplinary field aimed at assessing the condition, safety, and performance of engineering structures throughout their service life [42]. Traditional approaches to structural assessment relied on periodic visual inspections and localized non-destructive testing (NDT) techniques [6]. Although these methods are still valuable for identifying visible defects, they are inherently limited in their ability to detect hidden damage, capture time-dependent degradation, or provide early warning of incipient failures [7].

The increasing scale, complexity, and societal role of modern infrastructure have exposed the limitations of traditional inspection methods [8]. This has driven a move toward continuous, sensor-based monitoring systems. In this framework, SHM signifies a shift from reactive condition assessments to proactive and predictive management of infrastructure.

In recent years, SHM has become a core component of resilience and sustainability in infrastructure management. Modern SHM approaches go beyond damage detection to encompass performance evaluation, risk assessment, prognosis, and lifecycle optimization, enabling informed decisions amid uncertainty and evolving operational scenarios.

### 3.2. SHM System Architecture and Functional Layers

**Figure 7:**
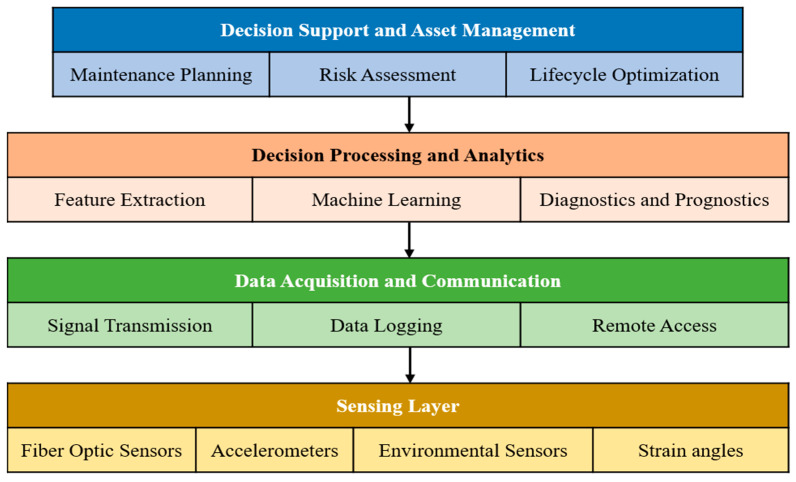
Generic architecture of an SHM system.

SHM systems are typically considered integrated architectures that combine sensing hardware, data acquisition and communication, data processing and interpretation, and decision-support mechanisms [43]. Unlike isolated inspection or measurement campaigns, SHM systems aim to interpret structural behavior holistically by analyzing spatially and temporally distributed data streams collected under operational conditions [44]. Figure 7 illustrates the generic architecture of an SHM system and its information flow. The framework illustrates a bottom-up progression from the sensing layer to decision support and asset management. Raw structural and environmental responses are first captured by heterogeneous sensors (e.g., fiber optic sensors, accelerometers, and environmental sensors), transmitted and logged through the data acquisition and communication layer, and subsequently processed using feature extraction, ML, and diagnostic/prognostic analytics.

The resulting information supports higher-level decision-making tasks, including maintenance planning, risk assessment, and lifecycle optimization. Upward arrows indicate the flow of information and knowledge from physical measurements to analytics-driven decision support. Overall, the figure emphasizes the end-to-end integration of sensing technologies, data analytics, and decision support, and provides a conceptual foundation for the subsequent discussion of fiber optic sensing technologies (Section 4) and ML-enabled intelligence (Section 6).

### 3.3. Fundamental Concepts in SHM

SHM systems are designed to capture damage-sensitive physical responses while remaining robust to benign variability arising from environmental and operational conditions [44]. Depending on the monitoring objective, SHM implementations are termed as global or local. Global SHM focuses on system-level behavior, often leveraging vibration-based or distributed sensing techniques. Whereas local SHM targets critical components or known damage-prone regions using high-resolution measurements of strain, displacement, or crack development [45]. SHM strategies may further be classified as continuous or periodic. Continuous monitoring is particularly valuable for safety-critical, dynamically loaded, or inaccessible structures, providing near-real-time information on structural conditions. Periodic monitoring remains appropriate for systems with relatively stable loading or limited sensing access, and hybrid strategies are frequently adopted in practice to balance cost, spatial coverage, and reliability [46].

From a functional standpoint, SHM aims to progress through increasing levels of damage identification, including damage detection, localization, severity assessment, and prognosis [47]. Higher diagnostic levels can be achieved through dense, reliable sensing, effective mitigation of environmental and operational influences, and advanced data interpretation frameworks [44]. From a quantitative perspective, many SHM frameworks formalize damage detection by comparing the current structural response to a reference or baseline representing a healthy state [48]. This comparison is commonly expressed through a normalized damage index that captures deviations in measured responses due to stiffness loss, cracking, or other degradation mechanisms. A generic representation of such a damage indicator can be written as [49]:

(1) $${DI}_{i}=pi=\frac{k_{i}^{u}-k_{i}^{d}}{k_{i}^{u}}$$

Here, $k_{i}^{u}$ and $k_{i}^{d}$ denote the stiffness of element *i* in the undamaged and damaged states, respectively. With this definition, *DI_i_* = 0 indicates no damage, and 0 < *DI_i_* < 1 represents stiffness loss (damage severity) in element *i*. While simple in form, this representation highlights the central challenge of SHM, distinguishing damage-induced changes from benign variations caused by environmental and operational conditions.

When effectively integrated into infrastructure management frameworks, SHM provides substantial lifecycle benefits. These include early identification of abnormal behavior, reduced reliance on manual inspections, optimized maintenance scheduling, and improved safety and resilience [17]. Moreover, long-term monitoring data provide valuable insight into the actual structural behavior under operational conditions, supporting model validation, performance assessment, and informed decision-making throughout the infrastructure life cycle [50].

### 3.4. Influence of Environmental and Operational Conditions

A persistent challenge in SHM is the influence of environmental and operational conditions (EOCs) on measured structural responses. Variations in temperature, humidity, traffic load, wind, wave action, and operational state can induce changes in sensor signals unrelated to structural damage [51]. In long-term monitoring deployments, EOC-induced variability may dominate the measured response, masking damage-sensitive features or leading to false alarms if not properly addressed. Strain- and vibration-based SHM data are particularly sensitive to temperature variations and operational loading. As a result, effective SHM frameworks must incorporate strategies to decouple damage-related responses from environmental influences. These strategies include multi-parameter sensing, statistical normalization, physics-based compensation models, and data-driven approaches [52]. The challenge posed by EOCs has been widely recognized as a key barrier to reliable, long-term SHM and a major motivation for advanced sensing configurations and intelligent data interpretation methods.

### 3.5. SHM Strategies: Model-Based, Data-Driven, and Hybrid Approaches

SHM strategies are typically divided into model-based and data-driven methods. Model-based SHM uses physics-based or numerical models to simulate how structures behave and compares these predictions with actual measured data. While these approaches provide valuable physical insights into damage mechanisms, they can also be sensitive to uncertainties in modeling, simplifications, and unknown boundary conditions [53]. Data-driven SHM strategies derive insights directly from measured data through statistical analysis or ML methods. These approaches are especially appealing for complex structures or extensive monitoring systems where creating precise models is difficult. However, purely data-driven techniques often require large amounts of high-quality data and may struggle to perform well when environmental conditions change [54].

Recent studies increasingly emphasize hybrid SHM strategies that combine physical understanding with data-driven intelligence. These approaches incorporate physics-based constraints into learning algorithms or employ models to support feature selection and interpretation, thereby enhancing robustness, interpretability, and scalability [55]. To clarify the differences and practical implications of these SHM strategies, especially in relation to modern fiber optic sensing systems, Table 2 summarizes their main characteristics, strengths, and limitations.

### 3.6. Sensing Requirements for Modern SHM Systems

Sensing systems are the core of SHM frameworks because they directly influence the quality, resolution, and reliability of the collected data. An effective SHM sensing system requires sufficient sensitivity to detect damage-related changes, long-term stability, and reliable operation in harsh environmental conditions [44]. Additionally, sensing systems should have adequate spatial coverage to monitor both global and local structural behaviors. Modern SHM architectures increasingly favor networked, distributed sensing systems that can monitor large, complex infrastructures [59]. Moreover, these sensing requirements have driven growing interest in advanced sensing technologies capable of supporting long-term, large-scale, and intelligent SHM systems. Among these, FOS technologies are well-suited to address the challenges of spatial coverage, durability, environmental robustness, and data richness, providing a natural transition to the detailed discussion in the following sections [60]. Building on this conceptual foundation of SHM systems and sensing requirements, the next section describes the systematic literature review methodology adopted to identify, screen, and synthesize research on FOS-based SHM and its integration with ML.

## 4. FOSs for SHM

### 4.1. Historical Development of FOSs

The development of FOSs is closely tied to advances in optical fiber communication technologies that emerged in the late 20th century. While optical fibers were initially designed for high-capacity signal transmission, early studies showed that external factors, such as strain, temperature, pressure, and vibration, affect light propagation within the fiber [61]. This observation laid the foundation for using optical fibers not only as communication media but also as intrinsic sensing elements. Early fiber-optic sensing approaches primarily relied on intensity-based and interferometric techniques. Although these methods demonstrated high sensitivity, their practical deployment was often constrained by signal instability, susceptibility to power fluctuations, and the complexity of interrogation systems [62]. A breakthrough occurred with the introduction of wavelength-encoded sensing, most notably FBG sensors, which offered improved measurement stability, repeatability, and multiplexing capability. These advantages significantly accelerated the adoption of FOSs in engineering and SHM applications [63].

**Figure 8:**
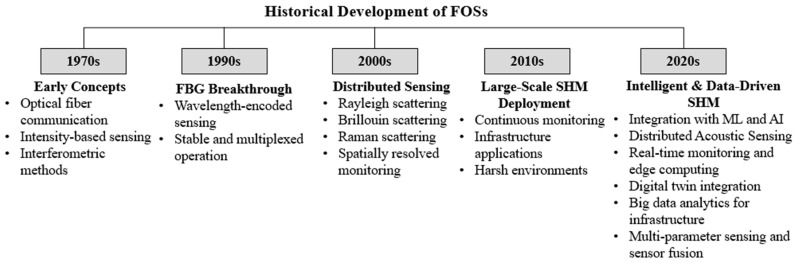
Historical evolution of FOS technologies from early communication-based concepts in the 1970s to modern large-scale SHM applications.

Subsequent progress in DFOS technologies based on Rayleigh, Brillouin, and Raman scattering further expanded sensing capabilities by enabling continuous, spatially resolved measurements along the full length of an optical fiber [64]. Collectively, these developments transformed FOSs into a versatile sensing platform capable of supporting large-scale, long-term SHM in complex, harsh environments, as documented in numerous comprehensive reviews. To provide a clearer perspective on how fiber optic sensing has evolved over time, Figure 8 summarizes the major milestones in the historical development of FOS technologies, highlighting the transition from early optical communication concepts to modern large-scale SHM applications.

Figure 8 illustrates how FOS technologies have evolved from primarily signal transmission applications to more robust, scalable sensing platforms. The shift became significant in the 1990s with the advent of wavelength-encoded sensing, which enhanced stability and multiplexing. Later, in the 2000s, advances in distributed sensing mechanisms enabled continuous, spatially detailed monitoring, paving the way for large-scale infrastructure SHM deployments in recent years.

### 4.2. General Principles of FOSs

FOSs operate by detecting changes in the optical properties of an optical fiber, such as intensity, phase, polarization, or wavelength, in response to external physical or environmental stimuli [64]. Depending on the sensing mechanism, these changes can be related to parameters including strain, temperature, pressure, vibration, and chemical exposure. A defining characteristic of fiber-optic sensing is the flexibility of sensing configuration, allowing measurements at discrete locations or continuously along the fiber length. This dominant characteristic enables adaptable monitoring strategies for a wide range of SHM applications [62].

Compared with conventional electrical sensors, FOSs offer several inherent advantages that are particularly relevant for SHM. These include protection from electromagnetic interference, resistance to corrosion, low weight, and the ability to transmit signals over long distances with minimal loss. As a result, FOSs are suitable for monitoring large-scale, distributed, and safety-critical structures where traditional sensing technologies may be limited [65]. Recent systematic reviews of advanced sensing technologies identify fiber-optic sensing as a promising technology for next-generation SHM frameworks. This is suitable for its scalability, compatibility with distributed sensing architectures, and long-term deployment in harsh environments.

### 4.3. Sensing Location–Based Categories of Fiber Optic Sensors

From an SHM perspective, FOSs are classified by the spatial distribution of sensing locations along the optical fiber. Based on this criterion, FOS technologies are generally categorized into point-based, quasi-distributed, and fully distributed sensing systems. This classification provides a practical framework for understanding the sensing philosophy, spatial resolution, data characteristics, and suitability of different fiber-optic technologies for specific monitoring objectives and infrastructure scales [66].

#### 4.3.1. Point Fiber Optic Sensors

Point fiber-optic sensors measure physical quantities at discrete, predefined locations along an optical fiber. Among available fiber-optic sensing technologies, FBG sensors are the most widely used for SHM applications [67]. An FBG sensor consists of a periodic modulation of the refractive index inscribed within the fiber core, which reflects a narrow band of wavelengths known as the Bragg wavelength. Strain or temperature variations modify the grating period and the effective refractive index, resulting in a measurable shift in the reflected wavelength [68].

Owing to their compact size, high sensitivity, multiplexing capability, and wavelength-encoded output, FBG sensors provide stable, long-term measurements and have been widely deployed in both laboratory investigations and real-world infrastructure monitoring. The sensing principle of an FBG is governed by the Bragg condition, which relates the reflected wavelength to the effective refractive index of the fiber core and the grating period [69].

(2) $$\lambda_{B}=2n_{eff}\Lambda$$

where *λ_B_* is the Bragg wavelength, *n_eff_* is the effective refractive index of the fiber core, and *Λ* denotes the grating period. Changes in strain or temperature alter these optical properties, resulting in a shift in the reflected Bragg wavelength, which underlies quantitative sensing [67].

In practical SHM deployments, both mechanical strain and temperature variations influence the reflected Bragg wavelength. Under linear assumptions, the combined effect of these parameters can be expressed as [70]:

(3) $$\Delta \lambda_{B}=\lambda_{B}\left[(1-p_{e})\varepsilon +(\alpha +\xi )\Delta T\right]$$

Here, *Δλ_B_* represents the Bragg wavelength shift, and *λ_B_* is the initial (unstrained) Bragg wavelength. The parameter *p_e_* is the effective photo-elastic coefficient of the optical fiber, accounting for strain-induced changes in refractive index. The variable ε denotes the axial mechanical strain at the grating location. The coefficient *α* represents the thermal expansion of the fiber material, while *ξ* is the thermo-optic coefficient describing temperature-induced changes in refractive index. *ΔT* denotes the temperature variation relative to a reference state. This formulation shows that Bragg wavelength shifts arise from both mechanical and thermal effects, underscoring the need for temperature-compensation strategies when FBG sensors are used for accurate strain-based SHM.

#### 4.3.2. Quasi-Distributed FOSs

Quasi-distributed fiber-optic sensing systems represent an intermediate step between single-point sensors and fully distributed sensing technologies. In most practical implementations, these systems consist of multiple FBG sensors multiplexed along a single optical fiber, each reflecting a distinct Bragg wavelength [71]. This configuration allows spatially discrete measurements at multiple locations while retaining the advantages of point sensors, including high sensitivity and measurement stability.

Compared with conventional electrical sensor networks, quasi-distributed FOS systems significantly reduce cabling complexity while providing greater spatial coverage than isolated point sensors. However, they do not provide continuous spatial information along the fiber length and require prior identification of monitoring locations [72]. As a result, quasi-distributed sensing is most effective in SHM applications where damage-prone regions are known in advance, such as bridge girders, tunnel linings, structural joints, and composite components.

#### 4.3.3. Distributed Fiber Optic Sensors

DFOSs are the advanced class of fiber-optic sensing technologies for SHM, enabling continuous spatial monitoring along the entire length of an optical fiber [73]. DFOS systems exploit naturally occurring fiber scattering to interrogate thousands of sensing points along a single sensing line. Unlike point-based or quasi-distributed systems, DFOS technologies rely on variations in the backscattered optical signal along the fiber to obtain spatially resolved measurements. In Rayleigh- and Brillouin-based DFOS technologies, local changes in strain and temperature induce measurable shifts in the backscattered signal, which can be approximated as [66,74].

(4) $$\Delta v\left(z\right)=C_{\varepsilon }\varepsilon \left(z\right)+C_{T}\Delta T(z)$$

where *Δν(z)* denotes the measured frequency (or spectral) shift at position *z* along the optical fiber, *ε(z)* is the axial strain field, and *ΔT*(*z*) represents the temperature change at location *z*. The coefficients *C_ε_* and *C_T_* are the strain and temperature sensitivity coefficients of the sensing mechanism, respectively, and depend on the underlying scattering process (e.g., Rayleigh or Brillouin). This formulation emphasizes the continuous, spatially distributed sensing capability of DFOS technologies, enabling simultaneous measurement of strain and temperature over the entire fiber length [75]. Such characteristics are particularly advantageous for monitoring large-scale, linear infrastructure systems, including pipelines, tunnels, railways, pavements, and other extended assets.

DFOS technologies rely on distinct light–matter interaction mechanisms. Brillouin-based sensors offer sensing ranges of tens of kilometers and are suitable for large-scale infrastructure such as pipelines and tunnels, where extensive spatial coverage is required. Raman-based sensors are primarily temperature-sensitive and are commonly used in fire detection and leak monitoring. These harsh conditions lead to abnormal thermal signatures that indicate potential faults. Rayleigh-based distributed fiber optic sensing techniques, such as optical time-domain reflectometry (OTDR) and optical frequency-domain reflectometry (OFDR), provide high spatial resolution for detecting localized strain variations, crack initiation, and minor damage. These methods are based on fundamental light-scattering principles and are not limited to a specific manufacturer or instrument [76,77]. Due to their scalability, dense spatial coverage, and ability to capture system-wide responses, DFOS technologies play a central role in next-generation SHM systems for large-scale, linear infrastructure, including pipelines, tunnels, railways, pavements, and long-span bridges [78,79,80].

In addition to traditional distributed sensing techniques based on Rayleigh, Brillouin, and Raman scattering, Distributed Acoustic Sensing (DAS) based on phase-sensitive optical time-domain reflectometry (Φ-OTDR) has become a powerful method for capturing high-frequency dynamic responses. Unlike conventional DFOS systems, which mainly monitor quasi-static parameters such as strain and temperature, DAS enables the detection of transient events, including vibration, impact, and acoustic emissions, along the entire length of the sensing fiber [81]. These dynamic signals carry detailed information about the initiation and progression of structural damage, making DAS especially useful for applications such as crack detection, intrusion monitoring, and vibration-based structural assessment [82]. Additionally, the combination of DAS measurements with advanced deep learning models has attracted significant attention, as data-driven approaches are effective at uncovering complex patterns in high-frequency acoustic signals. Consequently, DAS-based monitoring is a rapidly evolving and important direction in fiber-optic sensing-enabled SHM systems.

Table 3 summarizes point-based, quasi-distributed, and fully distributed FOS systems by representative technologies, spatial coverage, resolution, advantages, limitations, and typical applications. This classification clarifies why DFOS systems differ fundamentally from point-based and quasi-distributed approaches in sensing philosophy, data characteristics, and monitoring capability for extended infrastructure systems.

While Table 3 focuses on the classification and functional characteristics of fiber-optic sensing configurations, Table 4 provides a direct comparison of conventional SHM sensors and FOSs across critical performance attributes. These include durability, spatial coverage, environmental robustness, scalability, and compatibility with intelligent, data-driven SHM frameworks. This comparison highlights the fundamental limitations of traditional sensing approaches. It underscores the technical motivations driving the increasing adoption of fiber optic sensing DFOS technologies for large-scale, long-term, and intelligent SHM applications, particularly.

These advantages position DFOS technologies as a foundational sensing layer for the ML-enabled SHM frameworks discussed in the next Section.

The characteristics of signals generated by different fiber-optic sensing techniques, including their dimensionality, spatial resolution, and temporal behavior, directly influence the selection of appropriate data processing and machine learning strategies in SHM applications.

## 5. FOS Applications in SHM

FOSs have been widely adopted across diverse infrastructure sectors because they provide high-resolution, long-term, and spatially distributed measurements under harsh environmental conditions. Building on the sensing principles and technologies discussed in Section 3, this section synthesizes the use of FOS-based SHM systems across civil, marine, aerospace, energy, and transportation infrastructures, with an emphasis on monitored parameters, deployment strategies, and application-specific challenges. Comprehensive reviews and domain-focused studies demonstrate that fiber-optic sensing has progressed from experimental deployments to mature monitoring solutions in many real-world systems [14,15,31].

For infrastructure monitored using distributed fiber-optic sensing, the measured response is inherently spatiotemporal. Instead of isolated point measurements, DFOS provides continuous strain or temperature profiles along the structure [50]. This field-based nature of the data is particularly compatible with advanced data analytics and ML-based interpretation to identify damage localization, detect anomalies, and recognize patterns in complex infrastructure systems.

### 5.1. Civil Infrastructure Applications

#### 5.1.1. Bridges

Bridges are among the most extensively investigated civil infrastructure applications of fiber-optic-based SHM [102,103]. Both FBG sensors and DFOS systems have been used to monitor strain, deflection, vibration, temperature effects, and fatigue-related damage in steel, concrete, and composite bridge structures [104]. In particular, distributed Rayleigh- and Brillouin-based sensing techniques are used for continuous spatial monitoring of long-span bridges, capturing strain distributions along girders, decks, and cables that are difficult to obtain with conventional point-based instrumentation [45,105].

As bridges are exposed to harsh environmental conditions, FOSs are the best choice for long-term monitoring due to their durability, invulnerability to electromagnetic interference, and resistance to environmental degradation [88]. Many modern studies endorsed their effectiveness in early damage detection, validation of design assumptions, and support of condition-based maintenance and asset management strategies [106,107].

#### 5.1.2. Buildings

In building structures, FOSs have been used to monitor structural deformation, load redistribution, crack development, and temperature-induced responses under both operational and extreme loading conditions. FBG-based systems are commonly embedded in reinforced concrete elements or surface-mounted on structural members to provide localized strain measurements. In contrast, DFOS systems enable distributed monitoring across walls, slabs, and columns [108].

Applications in high-rise buildings and seismically active regions have demonstrated the suitability of FOSs for long-term monitoring and post-event damage assessment. In such environments, conventional electrical sensors may experience wiring damage, signal drift, or data loss, whereas fiber-optic systems maintain reliable performance under demanding conditions [109,110].

#### 5.1.3. Tunnels

SHM of tunnels has benefited significantly from the adoption of distributed fiber-optic sensing technologies. DFOS systems are widely used to monitor strain evolution, convergence, lining deformation, and crack initiation along tunnel linings during both the construction and operational phases [6,73]. The continuous spatial coverage provided by DFOS is particularly advantageous in tunnel environments, where damage locations are often unknown in advance and may develop over time.

Recent state-of-the-art reviews emphasize that fiber-optic sensing enables early detection of localized damage and supports long-term condition assessment of tunnel structures, contributing to improved safety management and maintenance planning [20,80].

#### 5.1.4. Dams

Fiber-optic sensors have also been widely deployed in dam monitoring to measure strain, temperature gradients, seepage-related effects, and overall structural deformation. Both FBG sensors and DFOS systems have been embedded within concrete dams to provide internal measurements that are difficult to obtain with surface-mounted instrumentation [33,111].

Long-term monitoring studies indicate that fiber-optic sensing enhances understanding of dam behavior under thermal loading, reservoir-level fluctuations, and aging. These insights support improved safety assessment, early anomaly detection, and risk-informed decision-making for dam operation and management [112].

### 5.2. Marine and Offshore Structures

#### 5.2.1. Ships

On marine vessels, FOSs are used to monitor hull strain, vibration, fatigue damage, and temperature under highly corrosive, dynamically varying operating conditions. FBG sensors are particularly well-suited for ship structures because of their compact size, ease of integration, and inherent resistance to electromagnetic interference, which is critical in electrically noisy onboard environments [42,113]. The existing literature emphasizes the effectiveness of fiber-optic sensing for fatigue monitoring and structural integrity assessment under wave-induced and operational loading, supporting condition-based maintenance of marine vessels [114].

#### 5.2.2. Offshore Platforms

Offshore platforms are exposed to severe environmental conditions and complex loading, including wave action, wind, currents, and operational loads, making them strong candidates for fiber-optic-based SHM. DFOS systems are widely used to monitor strain, temperature, and vibration in critical components such as platform legs, risers, and topside structures [115]. The ability of DFOS technologies to deliver long-range, continuous measurements along structural elements helps identify localized damage, deformation, and corrosion that conventional inspection methods may miss. Numerous studies in marine SHM indicate that adopting fiber-optic sensing improves monitoring reliability and can reduce inspection frequency and operational costs in offshore environments [12].

#### 5.2.3. Underwater Pipelines

Subsea pipelines are among the most well-established applications of distributed fiber-optic sensing technologies. These are routinely used to monitor strain evolution, temperature anomalies, seabed movement, and leak-induced thermal signatures along pipeline routes [115]. Due to their continuous spatial coverage and long sensing range, fiber-optic systems are effective in subsea environments for overcoming limited access and the cost and impracticality of conventional inspection [116,117]. Studies on subsea pipeline monitoring indicate that fiber-optic sensing helps early leak detection, supports real-time integrity assessment, and enhances risk-informed pipeline management in challenging underwater conditions [118,119].

### 5.3. Aerospace Structures

#### 5.3.1. Aircraft and Spacecraft

FOSs have been extensively investigated for SHM of aircraft and spacecraft due to their lightweight design, high sensitivity, and reliability under demanding service conditions [8]. FBG sensors are commonly embedded in composite airframes to monitor strain, damage accumulation, and temperature during manufacturing and in-service operation [51]. Their compatibility with composite materials and resistance to electromagnetic interference make them particularly attractive for aerospace applications.

The latest studies demonstrated the integration of fiber-optic sensing technologies with ML techniques for damage detection, classification, and prognosis in aerospace structures [35]. Such approaches are especially relevant for composite components, where damage mechanisms are often complex and challenging to detect using conventional inspection methods [120].

#### 5.3.2. Satellites

In satellite systems, FOSs are used to monitor structural deformation, thermal gradients, and vibration under extreme temperature cycling and radiation exposure. To address these harsh conditions, specialized FBG sensors and radiation-hardened optical fibers have been developed to ensure long-term measurement stability in space environments [35]. Historical and contemporary reviews from space agencies and research institutions emphasize the growing role of fiber-optic sensing in spacecraft engineering, including structural qualification, launch monitoring, and in-orbit condition assessment [121,122].

### 5.4. Energy Infrastructure

#### 5.4.1. Wind Turbines

FOSs have been widely used on wind turbine blades and towers to monitor strain, vibration, fatigue damage, and temperature under cyclic, highly variable loading conditions. DFOS ensures continuous measurements along the blade length, providing valuable insight into load distribution, damage initiation, and performance degradation that conventional sensors cannot capture [123,124,125]. These capabilities support both structural integrity assessment and operational optimization of wind energy systems.

#### 5.4.2. Nuclear Power Plants

In nuclear power facilities, FOSs are utilized for their resistance to electromagnetic interference and their ability to operate in extreme temperature, radiation-prone environments. Reported applications include monitoring structural components, containment systems, and temperature fields in safety-critical areas [126]. The fiber-optic sensing offers a robust alternative to conventional instrumentation in nuclear settings, where sensor reliability and long-term stability are essential for safe operation [127,128].

#### 5.4.3. Oil and Gas Pipelines

Oil and gas pipelines are among the most extensively monitored energy infrastructure using fiber-optic sensing technologies. DFOS systems are used for strain monitoring, leak detection, third-party intrusion detection, and temperature profiling along pipeline corridors [129]. The pipeline-focused reviews show that these have evolved into a cornerstone technology for pipeline integrity management, enabling continuous surveillance, early fault detection, and improved risk mitigation across large-scale transmission networks [69,130].

### 5.5. Transportation Infrastructure

#### 5.5.1. Roads and Highways

In roadway and pavement monitoring, FOSs have been embedded in pavement layers to measure strain, temperature, traffic-induced responses, and progressive structural degradation. Existing applications demonstrate the potential of fiber-optic sensing for continuous traffic monitoring, load assessment, and long-term pavement performance evaluation under real operating conditions [64,131]. These capabilities support data-driven pavement management and infrastructure planning.

#### 5.5.2. Railways

SHM of railway infrastructure has increasingly adopted DFOS to measure rail strain, track deformation, axle loads, and vibration over extended distances. These systems enable continuous monitoring of rail tracks, supporting early detection of defects, abnormal loading conditions, and degradation trends, thereby informing maintenance planning and operational safety [132]. Recent studies consistently emphasize the advantages of fiber-optic sensing for railway applications, particularly in terms of scalability, robustness, and suitability for long-distance monitoring [106,133]. Table 5 summarizes FOS applications across major infrastructure domains, highlighting commonly used sensing technologies, monitored parameters, key advantages and challenges, and their readiness for ML-based interpretation.

## 6. ML for Intelligent FOS-Based SHM

The integration of ML with FOS-based SHM reflects a broader shift away from simply collecting large volumes of monitoring data toward the development of more intelligent, automated decision-support systems [13,31]. As discussed in Section 3 and Section 4, modern fiber-optic sensing technologies, particularly quasi-distributed FBG arrays and distributed systems, continuously capture high-resolution strain, temperature, and vibration data across extensive structural regions. This level of detail significantly improves the ability to observe structural behavior in real time. At the same time, however, it introduces new challenges, including high data dimensionality, nonlinear structural responses, environmental influences, and difficulties in interpreting complex results. In many cases, these issues cannot be adequately addressed using traditional signal-processing methods or simple threshold-based analysis.

From an ML perspective, SHM using FOSs can be viewed as a data-driven process that links measured sensor responses to indicators of structural condition, damage state, or future performance [15]. This concept provides a unifying framework for a wide range of SHM objectives, including damage detection, localization, severity assessment, and prognosis, across diverse sensing configurations and infrastructure types [51]. However, extracting reliable and actionable information from FOS data requires learning algorithms capable of handling large-scale, heterogeneous, and often noisy datasets.

**Figure 9:**
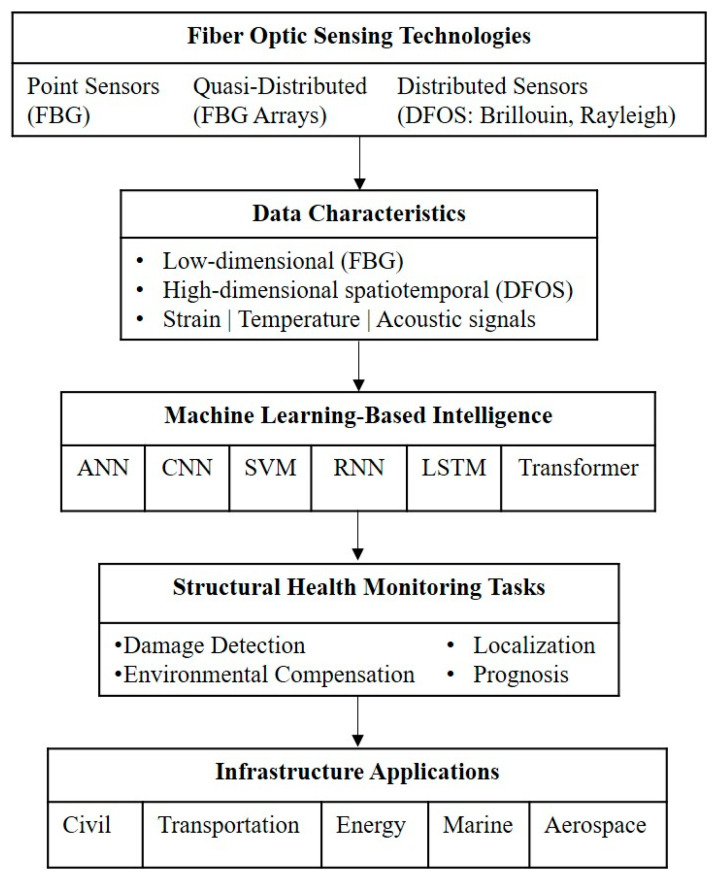
Conceptual framework showing the integration of fiber-optic sensing technologies, data characteristics, and machine learning models.

ML offers a robust set of tools to address these challenges by directly learning complex, nonlinear relationships from sensor data [134]. ML techniques enable automated feature extraction, anomaly detection, damage localization, compensation for environmental effects, and predictive assessment in SHM systems [135]. When combined with fiber-optic sensing, ML serves as a critical link between raw optical measurements and actionable engineering insights. Accordingly, this section discusses ML approaches from a task-oriented SHM perspective rather than focusing on individual algorithms in isolation. Emphasis is placed on how different learning paradigms align with the characteristics of fiber-optic sensing data and with the practical objectives and constraints of real-world SHM systems. To further clarify the integrated relationship between fiber-optic sensing technologies, data characteristics, machine learning models, and structural health monitoring objectives, a unified conceptual framework is presented in Figure 9.

Unlike conventional descriptive reviews, this framework explicitly illustrates how different sensing configurations generate distinct data structures, which in turn influence the selection of appropriate machine learning models and their alignment with specific SHM tasks. This task-oriented integration provides a system-level perspective linking sensing physics with data-driven intelligence across multiple infrastructure domains.

As shown in Figure 9, point-based and quasi-distributed sensing systems typically produce low-dimensional data that are well-suited to classical machine learning models such as ANN and SVM. In contrast, distributed fiber-optic sensing systems generate high-dimensional spatiotemporal data streams, requiring more advanced architectures such as CNNs, RNNs, LSTM networks, and Transformer models to effectively capture spatial patterns and temporal dependencies.

This framework also highlights the progression from raw sensing data to higher-level SHM tasks, including damage detection, localization, environmental compensation, and prognosis. By explicitly linking sensing modalities, data characteristics, and learning paradigms, the framework addresses a key gap in the existing literature and provides a structured foundation for future research and practical implementation of intelligent SHM systems.

### 6.1. ML Tasks in FOS-Based SHM

**Figure 10:**
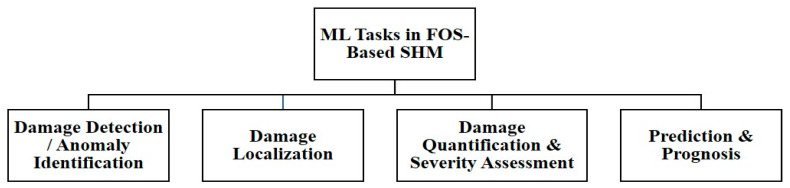
Functional categorization of machine learning tasks in fiber optic sensor-based SHM.

From a practical perspective, ML applications in FOS-based SHM can be grouped into four main tasks. First, ML models are used for damage detection, aiming to distinguish normal structural behavior from abnormal or damage-related responses. Second, once damage is detected, ML can help identify its location within the structure. Third, models can be trained to estimate the severity of the damage and its progression over time. Finally, ML can be used for prediction and prognosis, such as forecasting future structural performance or estimating the system’s remaining useful life. Figure 10 summarizes these four functional roles of machine learning in FOS-based SHM.

Each of these tasks places different demands on how the data are handled and how the models are designed. For example, point-based FBG sensors typically produce relatively compact feature sets that can often be handled with classical supervised learning methods. In contrast, DFOS systems generate spatial or space–time data fields, which are better suited to models that can capture spatial patterns and temporal trends, such as convolutional or sequence-based architectures. In practice, these task boundaries often overlap. Many monitoring systems combine detection, localization, and severity assessment within a single analytical pipeline. Table 6 summarizes these relationships from a task-oriented perspective, highlighting how different SHM goals align with particular FOS data types and model families, rather than focusing on individual algorithms in isolation.

Among the learning strategies summarized in Table 6, ANNs have been most widely adopted across multiple SHM tasks due to their flexibility and ability to model nonlinear relationships, motivating their discussion first.

Although the application of these sequence-based models in DFOS-specific studies remains relatively limited, their proven effectiveness in general structural health monitoring and time-series analysis strongly supports their potential for future integration with DFOS data. In particular, their ability to capture long-term temporal dependencies makes them well-suited for tasks such as damage prognosis, environmental compensation, and long-term performance monitoring in distributed sensing systems.

Before applying machine learning models, advanced signal preprocessing is critical to ensure reliable interpretation of fiber-optic sensing data. DFOS systems generate large volumes of high-dimensional, often noisy, spatiotemporal data, including non-stationary signals influenced by environmental and operational variability.

To address these challenges, various signal processing techniques are employed, including filtering methods, time–frequency analysis (e.g., wavelet transforms), feature extraction, and data fusion strategies. These approaches enable the transformation of raw sensing signals into compact, informative feature representations that are better suited for machine learning models.

Effective preprocessing not only improves model accuracy but also reduces computational complexity, particularly in real-time SHM applications and edge computing scenarios. By converting terabyte-scale raw data into lower-dimensional feature spaces, signal processing techniques help bridge the gap between sensing and intelligent decision-making in practical SHM systems.

These data characteristics fundamentally determine the suitability of different machine learning models, highlighting the need to align sensing physics with data-driven methodologies.

### 6.2. Artificial Neural Networks

ANNs are among the most widely adopted ML techniques in SHM due to their flexibility in modeling nonlinear relationships between sensor measurements and structural states [136]. In FOS-based SHM, ANNs have been extensively applied to tasks such as strain estimation, temperature compensation, damage classification, and system identification using data from both FBG sensors and DFOS systems [137].

ANN-based approaches are particularly useful in this context because they can accommodate noisy measurements, nonlinear sensor–structure interactions, and multivariate inputs typical of fiber optic sensing. However, their performance is strongly influenced by network architecture, training data quality, and robustness to environmental and operational variability. These factors are critical in long-term monitoring applications, where changing conditions and limited availability of labeled damage data can affect model generalization.

#### 6.2.1. Feedforward Neural Networks

Feedforward neural networks (FNNs) are the most basic ANN architecture, consisting of an input layer, one or more hidden layers, and an output layer, with unidirectional information flow [138]. In FOS-based SHM, FNNs are commonly used to map optical measurements such as FBG wavelength shifts or reduced DFOS feature sets to structural parameters, condition indicators, or damage-related metrics.

FNNs have been widely applied to strain estimation, temperature compensation, and structural response prediction due to their computational efficiency and ease of implementation. FNNs offer a balance between modeling capability and computational cost. This aspect makes them suitable for real-time or near-real-time monitoring when representative training data is available. However, their applicability to high-dimensional DFOS datasets is often limited unless combined with dimensionality reduction or feature extraction techniques.

#### 6.2.2. Backpropagation Neural Networks

Backpropagation Neural Networks (BPNNs) are a widely used training paradigm for FNNs, in which model parameters are optimized through gradient-based minimization of prediction error. BPNNs have been extensively applied in SHM for damage classification, anomaly detection, and parameter estimation using data from FBG arrays and DFOS systems [139,140].

Although many studies projected strong predictive performance, systematic reviews highlight several limitations of BPNNs. The worth mentioning are our susceptibility to overfitting, sensitivity to the quality of the training data, and reduced generalization across varying environmental and operational conditions. These challenges are gaining interest in regularization techniques, physics-informed constraints, and hybrid data-driven–physics-based modeling approaches to enhance robustness in long-term FOS-based SHM deployments.

### 6.3. Convolutional Neural Networks

CNNs have attracted increasing attention in FOS-based SHM because they can automatically extract hierarchical features from structured data [141]. CNNs are particularly well-suited for DFOS data represented as spatial profiles or spatio-temporal maps, such as strain distributions along pipelines, tunnels, and bridge decks.

**Figure 11:**
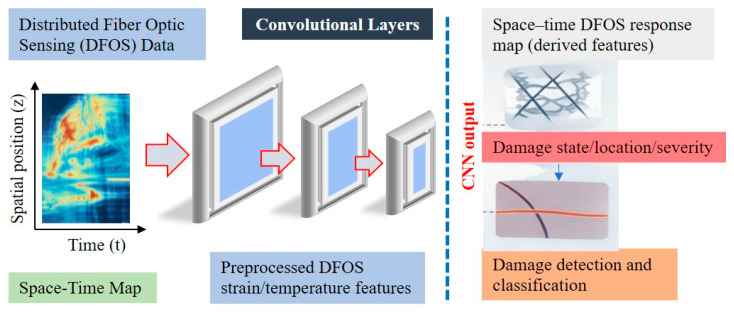
Conceptual CNN-based workflow for processing DFOS data in SHM.

By exploiting convolutional filtering and pooling operations, CNN-based models can effectively capture localized damage signatures embedded within high-dimensional DFOS datasets while reducing sensitivity to measurement noise and spatial variability [142]. Despite these advantages, the deployment of CNN-based approaches in safety-critical SHM applications is limited by data availability, computational requirements, and limited model interpretability. Addressing these challenges is an active area of research, with recent efforts focusing on explainable learning frameworks, transfer learning, and physics-informed neural networks to improve reliability and acceptance in real-world monitoring systems. Figure 11 presents the CNN-based workflow for DFOS-driven SHM, in which spatiotemporal response maps are processed by convolutional layers to enable automated damage detection, localization, and severity estimation.

DFOS systems produce high-resolution spatiotemporal data streams that record both spatial variations along the sensing fiber and the temporal evolution under dynamic loading conditions. While CNNs are effective at extracting spatial features, they are limited in capturing long-term temporal dependencies inherent in such data. Sequence-based models, including recurrent neural networks (RNNs), LSTM networks, and Transformer architectures, offer a more suitable framework for modeling these temporal dynamics.

LSTM and gated recurrent unit (GRU) models have been widely used in structural health monitoring for time-series prediction and damage progression analysis, enabling the capture of temporal correlations under varying operational and environmental conditions. More recently, Transformer-based models, which utilize self-attention mechanisms, have demonstrated strong performance in modeling long sequences and show promise for deformation prediction and structural condition assessment. Although their use in DFOS-based SHM remains limited, these architectures offer significant potential for managing large-scale spatiotemporal sensing data and enhancing the robustness of prognostic and anomaly-detection models.

### 6.4. Support Vector Machines

SVMs are supervised learning algorithms widely used in SHM for both classification and regression tasks. These have been applied to damage detection, condition classification, and damage severity assessment using features extracted from FBG arrays and DFOS measurements [143].

SVMs are particularly useful when labeled damage data are limited, as they often demonstrate strong generalization and robustness to measurement noise. These characteristics make them suitable for early-stage monitoring and baseline damage detection tasks. However, their application to large-scale DFOS datasets is typically constrained by computational scalability, sensitivity to kernel selection, and the need for dimensionality reduction when handling high-dimensional spatial or spatio-temporal data. As a result, SVM-based approaches are most effective when applied to reduced feature sets or localized monitoring regions rather than full-length distributed sensing data [144,145].

### 6.5. Other Machine Learning Algorithms

Beyond neural networks and SVMs, a range of additional ML-learning algorithms has been explored for intelligent fiber-optic sensor-based SHM. These include decision trees, random forests, k-nearest neighbors, Gaussian process regression, and ensemble learning methods [146]. Such techniques are commonly used for tasks such as feature selection, anomaly detection, regression-based damage assessment, and uncertainty quantification, often offering improved interpretability compared with deep learning approaches [147].

Also, many recent studies highlight a positive trend in unsupervised and semi-supervised learning strategies, particularly for long-term SHM deployments where labeled damage data are scarce or unavailable. In parallel, physics-informed and hybrid ML-learning models are emerging as promising solutions for integrating domain knowledge with data-driven methods.

### 6.6. Unified Learning Formulation for FOS-Based SHM

Most supervised ML approaches used in FOS-based SHM can be expressed within a unified optimization framework [148]:

(5) $$\hat{\theta }=arg\;\underset{\theta }{min}\sum_{i=1}^{N}L(f_{\theta }(x_{i}),y_{i})+\lambda \Omega (\theta )$$

where $x_{i}$ denotes the FOS measurements (e.g., FBG features or DFOS-derived representations), $y_{i}$ represents the corresponding structural condition labels or damage indicators, $f_{\theta }$ is the learned mapping parameterized by *θ*, *L* is an application-dependent loss function, and *Ω*(*θ*) is a regularization term that penalizes model complexity. The parameter *λ* balances between data fidelity and regularization. This formulation highlights the fundamental trade-off between fitting observed sensor data and controlling model complexity, a critical consideration in SHM applications with limited labeled damage data, environmental variability, and long-term monitoring requirements.

### 6.7. Key Challenges and Emerging Directions

Even with the progress made in recent years, there are still practical hurdles that slow down the use of ML-enabled, FOS-based SHM systems. One of the biggest issues is the lack of labeled damage data, since real damage events are rare and controlled experiments do not always reflect field conditions [31]. In addition, measurements are often influenced by temperature, loading changes, and other environmental effects, which can confuse models and reduce reliability. Model interpretability is another concern, especially for deep learning approaches that are difficult to explain in safety-critical applications [149]. It is also challenging to apply a model trained on one structure or material to a different site with different operating conditions.

To address these limitations, recent studies are moving toward hybrid and physics-informed learning approaches, closer integration with digital twins, and uncertainty-aware or probabilistic modeling strategies. These efforts aim to make models more robust, transparent, and adaptable in real-world settings. Table 7 summarizes the main challenges and outlines key research directions for advancing ML-enabled fiber-optic sensor-based SHM.

### 6.8. Challenges, Issues, and Future Research Directions

Although FOS-based SHM and its integration with ML have advanced considerably, several technical and practical challenges still require careful attention. Overcoming these issues is critical if such systems are to move beyond laboratory studies and pilot projects and become reliable, scalable tools for real infrastructure monitoring. In this subsection, the main challenges in sensing hardware, data handling, ML implementation, and system-level deployment are discussed, followed by key research directions to support the next stage of development in this field.

#### 6.8.1. Sensing and Hardware-Related Challenges

Although FOSs offer clear advantages, installing and maintaining them in real infrastructure systems is not always straightforward. In many cases, installation is particularly challenging when distributed systems must be retrofitted into existing structures. Concerns about sensor survival during construction, long-term durability in harsh environments, and protection against mechanical damage still limit wider field deployment.

For FBG-based and quasi-distributed systems, sensor placement and spacing require careful planning to balance spatial resolution, coverage, and overall cost. In DFOS, inherent trade-offs among sensing range, spatial resolution, acquisition speed, and signal quality make it difficult to simultaneously capture fast transient events and maintain long-distance coverage. Long-term calibration drift and strain–temperature cross-sensitivity also remain practical concerns in field applications.

Moving forward, improvement in this area will likely depend on better sensor packaging, more reliable installation practices for both new and existing structures, and advances in interrogation hardware that increase resolution and acquisition speed without sacrificing sensing range or reliability.

#### 6.8.2. Data Volume, Quality, and Management

Modern FOS-based SHM systems, especially those built around DFOS, produce large volumes of data that vary both in space and over time. Over extended monitoring periods, handling these datasets can become demanding in terms of storage capacity, data transfer, and computational resources. In practice, the challenge is not limited to data size alone. Measurement noise, signal attenuation over long distances, intermittent data gaps, and occasional sensor malfunctions all add layers of complexity to interpretation.

Another practical limitation is the lack of broadly accepted data standards and benchmark datasets. Because different studies use different preprocessing steps, evaluation metrics, and reporting formats, comparing results across publications can be difficult. Reproducibility also becomes a concern when datasets are not publicly available or when experimental setups are highly customized. Moreover, long-term monitoring rarely produces stationary data. Environmental exposure, loading patterns, and operational conditions evolve over time, meaning that models developed under one set of assumptions may gradually lose accuracy as conditions change.

Progress in this area will likely depend on pragmatic solutions. More efficient data compression strategies, greater use of edge or fog computing to preprocess data close to the sensor, and the development of shared datasets with agreed evaluation procedures would help create a more stable and transparent foundation for intelligent SHM research.

#### 6.8.3. Transfer Learning for Data Scarcity

A key challenge in FOS-based SHM is the lack of labeled damage data, especially under real-world conditions where failures are infrequent. Transfer learning offers a practical and effective solution by utilizing knowledge from related domains or simulated datasets. In SHM applications, transfer learning allows models trained on lab tests, simulations, or similar structures to be adapted to target systems with limited labeled data. This approach has been successfully used in broader SHM areas for anomaly detection, damage classification, and condition assessment. Its integration with fiber-optic sensing data is particularly promising, as it combines physics-based simulation outputs with field measurements to enhance model generalization. Therefore, transfer learning should be regarded not only as a future research possibility but also as an immediate, practical method to improve machine learning applications in data-scarce SHM environments.

#### 6.8.4. Challenges in Machine Learning Integration

Machine learning offers significant potential for making sense of complex FOS-based SHM data, but translating theory into practice is not always easy. Many commonly used supervised models depend on labeled examples of damage. In real infrastructure, however, actual damage events are rare, and laboratory experiments often fail to capture the full variability present in field conditions. As a result, models that perform well in controlled settings may not behave as reliably when deployed on operational structures.

Interpretability is another issue that cannot be overlooked. Deep learning models, while powerful, often provide limited insight into how predictions are generated. For engineers and decision-makers responsible for safety-critical systems, this lack of transparency can create hesitation. Regulatory bodies may also require clearer justification of model outputs before accepting automated diagnostic tools. In addition, models trained under specific environmental conditions or loading scenarios can struggle when exposed to new or evolving operating contexts.

To address these concerns, research is gradually shifting toward approaches that incorporate domain knowledge and reduce dependence on large labeled datasets. Physics-informed learning, transfer learning, and semi-supervised or unsupervised methods are increasingly being explored as practical alternatives. These strategies aim to improve robustness and adaptability, making ML-enabled SHM systems more suitable for long-term, real-world deployment.

#### 6.8.5. Environmental and Operational Variability

Separating genuine damage signals from normal environmental and operational influences remains one of the most persistent difficulties in SHM. Temperature fluctuations, humidity changes, traffic loading, wind, wave action, and routine operational cycles can significantly influence fiber optic measurements. In many cases, these effects are strong enough to obscure subtle, early-stage damage signatures.

Although data-driven compensation techniques have shown encouraging results, their effectiveness depends heavily on how the sensing system is configured and how the data are processed. In practice, relying on a single measurement parameter rarely provides enough information to confidently distinguish structural deterioration from normal variability. This limitation becomes more pronounced in long-term monitoring, where environmental conditions evolve over time.

A more reliable path forward is to incorporate multi-parameter sensing strategies. Combining strain, temperature, and other environmental measurements can provide a more complete picture of structural behavior. Integrating fiber-optic data with auxiliary sensors or external datasets further strengthens interpretation and supports more reliable long-term assessment.

#### 6.8.6. System-Level Integration and Scalability

A common limitation in the current literature is the tendency to focus on individual components of FOS-based SHM systems, whether sensing hardware, data processing techniques, or machine learning models, without fully addressing how these elements function together in operational settings. Laboratory-scale demonstrations often highlight technical feasibility, but deploying such systems on real infrastructure introduces additional layers of complexity. Issues related to system integration, interoperability between hardware and software platforms, routine maintenance, and long-term lifecycle management become central concerns.

Achieving scalable and reliable SHM requires looking at the system as a whole rather than as a collection of separate technologies. Sensing devices, data management pipelines, learning algorithms, and decision-support tools must operate cohesively within a structured architecture. Emerging technologies such as cloud-based monitoring platforms, cyber–physical system frameworks, and digital twins offer practical avenues for integrating these components. When implemented thoughtfully, these approaches can help SHM systems adapt to varying infrastructure conditions while maintaining reliability over extended service periods.

#### 6.8.7. Toward Intelligent and Autonomous SHM Systems

Looking ahead, the broader ambition of FOS-based SHM is to move toward monitoring systems that are not only data-rich but also capable of learning, adapting, and supporting informed maintenance decisions. The goal is not simply to collect more measurements, but to develop systems that can assist with early diagnosis, adapt to changing conditions, and support predictive maintenance strategies. Reaching this level of capability requires tighter integration between sensing hardware, physics-based understanding of structural behavior, and machine learning techniques. Treating these elements as separate layers is no longer sufficient.

Several areas deserve continued attention. Physics-informed and hybrid learning models that reflect the characteristics of fiber optic data can help bridge the gap between purely data-driven approaches and engineering knowledge. The development of shared benchmark datasets and clearer evaluation procedures would also strengthen comparability and transparency across studies. At the system level, closer alignment between SHM platforms, digital twins, and decision-support tools is likely to play a central role in practical deployment. In addition, advances in edge-based computing can support real-time monitoring over large infrastructure networks, while ongoing work on interpretability and uncertainty estimation remains essential for building confidence in automated diagnostics. Progress in these directions will help shift FOS-based SHM from promising research demonstrations toward reliable tools that contribute meaningfully to safer, more resilient, and more sustainable infrastructure management.

Overall, a comparative evaluation of existing approaches indicates that no single machine learning model is universally optimal. Instead, effective SHM solutions require alignment between sensing modality, data characteristics, and analytical objectives. This reinforces the importance of the task-oriented framework proposed in this study, which provides a structured basis for selecting appropriate methodologies across different infrastructure applications.

## 7. Conclusions and Research Outlook

Given the field’s broad, interdisciplinary nature, the discussion emphasizes integrative insights rather than exhaustive technical detail in each subdomain. The objective is to provide a structured understanding of how sensing technologies and machine learning approaches can be jointly leveraged for SHM applications.

Overall, this review explores the growing integration of fiber-optic sensing technologies and machine learning methods for structural health monitoring of modern infrastructure systems. Rather than discussing sensing hardware and data analytics separately, the paper has considered them together, since their interaction ultimately determines how SHM systems function in practice. This integrated perspective clarifies how optical measurements and data-driven models complement one another in real-world monitoring environments.

The discussion began with the fundamentals of SHM and the practical limitations associated with inspection-based practices and conventional electrical sensors, particularly when monitoring large, distributed, or safety-critical structures over long periods. In this environment, fiber-optic sensing provides several practical advantages. Point-based FBG sensors, quasi-distributed arrays, and fully distributed DFOS systems each offer different levels of spatial resolution and coverage. Reported applications show that these technologies perform reliably in harsh environments, resist electromagnetic interference, and support high-resolution measurements over extended distances. Distributed sensing systems enable continuous monitoring along long, complex assets, whereas FBG-based approaches remain effective when monitoring locations are predefined.

Applications across civil, transportation, marine, aerospace, and energy infrastructure reveal similar patterns. Fiber-optic sensors make it possible to capture detailed strain and temperature fields that are difficult to obtain using conventional instrumentation. At the same time, this improved observability introduces new practical challenges. The volume and complexity of data generated by distributed systems, particularly in long-term deployments, make interpretation increasingly demanding. Environmental variability, operational effects, and measurement noise complicate the extraction of damage-related information, exposing the limitations of purely threshold-based or traditional signal-processing methods.

As a result, machine learning methods have become important tools for transforming raw sensor data into usable engineering information. When organized according to SHM objectives such as damage detection, localization, severity estimation, and prognosis, it becomes evident that different learning approaches align differently with point-based and distributed sensing configurations. Although neural networks, SVMs, and related algorithms have demonstrated encouraging performance in laboratory and field studies, several obstacles remain. Limited labeled damage data, evolving environmental conditions, and concerns regarding interpretability and transferability continue to restrict broader deployment in safety-critical applications. While significant progress has been achieved, broader adoption of fiber-optic-sensor-based SHM systems will depend on continued field validation, improved data standards, and closer integration between sensing technologies and intelligent data analytics.

Looking ahead, further progress will depend less on isolated algorithm development and more on coordinated system-level integration. Approaches that embed physics-based constraints into learning models, establish shared benchmark datasets, and more closely couple SHM platforms with digital twin environments may help address current limitations. Advances in edge computing and real-time analytics are also likely to support more scalable monitoring architectures, particularly for geographically distributed infrastructure networks.

Taken together, fiber-optic sensing and machine learning provide a realistic pathway for advancing structural health monitoring practice. Continued collaboration among sensing specialists, structural engineers, and data scientists supported by long-term field validation will be essential to ensure that these methods mature into dependable tools for real-world infrastructure management.

While the scope of this review spans multiple domains, its primary contribution lies in synthesizing these areas into a unified framework that supports the design and deployment of intelligent SHM systems. Future studies may further deepen individual aspects, such as advanced sensing physics or specialized machine learning architectures, building upon the integrative perspective established in this work.

## Figures and Tables

**Table 1:** Keyword groups used in the literature search strategy.

Concept Group	Keywords
Fiber optic sensing technologies	fiber optic sensor, optical fiber sensor, fiber Bragg grating, FBG sensor, distributed fiber optic sensing, distributed optical fiber sensing, DFOS
SHM	SHM, infrastructure monitoring
ML and data-driven analysis	machine learning, deep learning, neural network, support vector machine, data-driven

**Table 2:** Comparison of model-based, data-driven, and hybrid SHM approaches.

SHM Approach	Core Principle	Typical Methods	Key Strengths	Main Limitations	Suitability for FOS Data
Model-based SHM [56]	Structural condition is inferred by comparing measured responses with predictions from physics-based models	Finite element models, analytical mechanics models, modal updating, residual-based damage indices	Strong physical interpretability; direct linkage to damage mechanisms; suitable for prognosis when models are accurate	Sensitive to modeling uncertainties, boundary conditions, and material assumptions; limited scalability for complex or evolving structures	Moderate—effective for point-based FBG data and low-dimensional measurements; less suited for large DFOS datasets
Data-driven SHM [57]	Structural condition is inferred directly from measured data using statistical or ML techniques	Statistical pattern recognition, neural networks, SVM, clustering, anomaly detection	Capable of capturing nonlinear behavior; scalable to large datasets; well-suited for complex and distributed sensing	Requires large, high-quality datasets; limited interpretability; sensitive to environmental and operational variability	High—particularly suitable for quasi-distributed and distributed FOS data with rich spatial–temporal information
Hybrid SHM [58]	Combines physics-based models with data-driven learning to exploit complementary strengths	Physics-informed ML, model-guided feature extraction, Bayesian updating, digital twins	Improved robustness and generalization; enhanced interpretability; reduced data requirements compared to purely data-driven methods	Increased system complexity; higher implementation effort; requires interdisciplinary expertise	Very High—well aligned with FOS-based SHM, especially DFOS systems under varying environmental and operational conditions

**Table 3:** Classification of fiber optic sensors based on sensing location.

Sensor Category	Typical Technologies	Spatial Coverage	Spatial Resolution	Key Advantages	Key Limitations	Representative SHM Applications
Point FOSs [83]	FBG, Fabry–Pérot interferometers	Discrete locations (point-based)	Very high (localized, mm–cm scale)	High sensitivity; wavelength-encoded output; excellent long-term stability; easy multiplexing; suitable for embedment	Limited spatial coverage; damage must be anticipated at sensor locations	Local strain and temperature monitoring of critical components, joints, girders, and composite elements
Quasi-DFOSs [72,84,85]	Multiplexed FBG arrays (WDM, TDM)	Multiple predefined locations along a single fiber	High at sensor locations; moderate overall coverage	Reduced cabling; scalable sensing along structural members; good compromise between resolution and system complexity	Requires prior knowledge of damage-prone regions; not continuous	Bridges, tunnel linings, building frames, and aerospace structures with known critical zones
DFOS [75,86,87]	Rayleigh (OTDR/OFDR), Brillouin (BOTDR/BOTDA), Raman scattering	Continuous sensing along the entire fiber length (up to tens of km)	Moderate to very high (cm to m, depending on technique)	Full spatial coverage; unknown damage localization; ideal for large and linear infrastructures; compatible with ML-based interpretation	Large data volume; complex signal interpretation; trade-offs between range, resolution, and acquisition speed	Pipelines, tunnels, railways, pavements, dams, offshore and subsea infrastructure

**Table 4:** Comparison of Conventional SHM Sensors and FOSs.

Attribute	Conventional SHM Sensors	FOSs	Representative References
Durability	Degradation due to corrosion, moisture, fatigue, and connector aging; frequent recalibration is often required.	High durability owing to corrosion resistance, chemical inertness, and absence of electrical components.	[70,88]
Spatial Coverage	Primarily point-based or sparsely distributed measurements; dense coverage requires extensive wiring.	Point, quasi-distributed, and fully distributed sensing enabling continuous spatial measurements.	[89,90,91,92]
Environmental Robustness	Susceptible to EMI, temperature drift, and harsh environmental exposure.	Intrinsically immune to EMI; suitable for high temperature, radiation, and corrosive environments.	[93,94]
Scalability	Limited scalability due to cabling, power requirements, and data acquisition constraints.	High scalability through multiplexing and distributed sensing over long distances.	[95,96]
Long-Term Deployment	Long-term stability is affected by drift and environmental degradation.	Stable long-term performance with minimal signal degradation.	[97,98,99]
Compatibility with Intelligent SHM	Limited spatial resolution constrains advanced data-driven analysis.	Rich spatial–temporal data is well-suited for ML-enabled SHM.	[100,101]

**Table 5:** FOS applications across major infrastructure domains.

Infrastructure Domain	Sensor Type	Monitored Parameters	Key Advantages	Key Challenges	Readiness for ML-Based Interpretation
Bridges	FBG; DFOS (Rayleigh, Brillouin, Raman)	Strain, deflection, vibration, temperature, fatigue	Long-term stability; dense spatial coverage; EMI immunity	Environmental temperature effects: large data volume	High—widely applied in damage detection and prognosis
Buildings	FBG; quasi-distributed FBG arrays	Strain, displacement, crack development, temperature	Embedment capability; localized monitoring of critical members	Installation complexity; sensor survivability	Moderate–High—effective for anomaly detection and condition assessment
Tunnels	DFOS (Rayleigh, Brillouin, Raman)	Lining strain, convergence, and crack initiation	Continuous spatial monitoring; suitability for unknown damage locations	Signal interpretation in heterogeneous materials	High—well suited for spatial ML-based analysis
Dams	FBG; DFOS (Rayleigh, Brillouin, Raman)	Strain, temperature gradients, and seepage-related effects	Internal sensing; long-term durability	Thermal–mechanical coupling; environmental effects	Moderate—often requires physics-informed ML approaches
Marine Structures (Ships)	FBG	Hull strain, vibration, fatigue	Corrosion resistance; compact sensing	Harsh marine environment; sensor protection	Moderate
Offshore Platforms	DFOS (Rayleigh, Brillouin, Raman); FBG	Strain, vibration, temperature	Long-range monitoring; suitability for harsh environments	Installation cost; data transmission constraints	Moderate–High
Subsea Pipelines	DFOS (Rayleigh, Brillouin, Raman)	Strain, temperature, leakage signatures	Kilometer-scale coverage; effective leak detection	Large data streams; noise management	High—strong adoption of ML-based analytics
Aircraft	FBG	Strain, damage accumulation, temperature	Lightweight; high sensitivity; embedment in composites	Certification requirements; redundancy constraints	High—strong integration with ML-based SHM
Satellites/Spacecraft	Radiation-hardened FBG	Thermal gradients, deformation, vibration	EMI immunity; operation in extreme environments	Radiation effects; calibration stability	Moderate
Wind Turbines	FBG; DFOS (Rayleigh, Brillouin, Raman)	Blade strain, fatigue, vibration	Distributed blade monitoring; high sensitivity	Rotational dynamics; data synchronization	High
Nuclear Facilities	FBG; DFOS (Rayleigh, Brillouin, Raman)	Temperature, strain, structural integrity	Radiation tolerance; suitability for safety-critical monitoring	Sensor longevity; access constraints	Moderate
Oil & Gas Pipelines	DFOS (Rayleigh, Brillouin, Raman)	Strain, temperature, intrusion, leakage	Mature technology; real-time, continuous monitoring	Data management: false positives	Very High
Roads & Pavements	FBG	Strain, temperature, traffic-induced response	Embedment feasibility; high sensitivity	Sensor survivability under traffic loading	Moderate
Railways	DFOS (Rayleigh, Brillouin, Raman); FBG	Rail strain, axle loads, vibration	Long-distance monitoring; scalability	Data interpretation complexity	High

Note: The selection of machine learning approaches is influenced by data dimensionality, noise characteristics, and availability of labeled datasets, which may impact model performance and generalization.

**Table 6:** Mapping of SHM tasks, FOS data characteristics, ML strategies, and challenges.

SHM Task	Fiber Optic Sensor (FOS) Data Type	Input Representation	Typical ML Strategy	Output Type	Key Challenges
Damage detection/anomaly identification	FBG, quasi-distributed FBG, DFOS (Rayleigh, Brillouin)	Time series; statistical features; frequency-domain indicators	Supervised and unsupervised classifiers (e.g., ANN, SVM, autoencoders)	Binary or multi-class condition state	Environmental variability; limited labeled damage data; false positives
Damage localization	DFOS (Rayleigh, Brillouin); dense FBG arrays	Spatial strain/temperature profiles; space–time maps	Spatial learning and clustering models (e.g., CNN-based, density-based)	Damage location (coordinate or segment)	High data dimensionality; spatial noise; heterogeneous materials
Damage quantification/severity assessment	FBG, quasi-distributed FBG, DFOS	Feature vectors, spatial gradients, and response maps	Regression-oriented learning (e.g., ANN-based, tree-based)	Damage severity index or continuous metric	Limited ground truth; model transferability
Structural response prediction	FBG, DFOS	Time series; multivariate sensor streams	Sequence and temporal learning models	Predicted structural response (e.g., strain, displacement, temperature)	Long-term drift; non-stationary behavior
Prognosis and remaining useful life (RUL)	Long-term FBG and DFOS monitoring data	Temporal degradation trends; latent features	Probabilistic and temporal prediction models	RUL estimate; failure probability	Scarcity of failure data; uncertainty quantification
Environmental and operational compensation	FBG; DFOS (multi-parameter sensing)	Multi-channel strain–temperature data	Multivariate regression and domain-adaptation models	Environment-compensated response	Coupled effects: sensor cross-sensitivity
System-level condition assessment	Hybrid FBG–DFOS networks	Fused spatial–temporal features	Ensemble and hybrid ML–physics frameworks	System-level health index or decision-support indicator	Data fusion; interpretability; scalability

**Table 7:** Challenges and key research directions for advancing ML-enabled FOS-based SHM.

Category	Key Challenges	Implications for SHM Practice	Future Research Directions
Sensing & Hardware	Sensor survivability during installation and long-term operation; strain–temperature cross-sensitivity; trade-offs between spatial resolution, sensing range, and acquisition speed (especially in DFOS).	Limits the reliability of long-term monitoring and affects data fidelity under harsh environmental conditions.	Advanced sensor packaging and encapsulation; multi-parameter sensing; next-generation interrogators with higher resolution and faster acquisition.
Data Volume & Management	Massive, high-dimensional spatio-temporal datasets from DFOS; noise, signal attenuation, missing data, and sensor faults; lack of standardized data formats.	Increases computational burden and complicates long-term data storage, transmission, and interpretation.	Edge and fog computing for on-site preprocessing; efficient data compression; standardized data schemas and open benchmark datasets for FOS-based SHM.
Labeled Data Scarcity	Limited availability of labeled damage data; rarity of real damage events; reliance on laboratory or simulated datasets.	Reduces robustness and generalization of supervised ML models in field deployments.	Semi-supervised, unsupervised, and self-supervised learning; simulation-augmented learning; transfer learning across structures and sites.
Environmental & Operational Variability	Dominance of temperature, humidity, and operational effects over damage-sensitive signals; non-stationary long-term data.	Leads to high false-alarm rates and reduced sensitivity to early-stage damage.	Multi-parameter sensing; physics-informed ML; hybrid compensation strategies combining physical models and data-driven approaches.
Model Interpretability	Black-box nature of deep learning models (e.g., CNNs, RNNs); limited physical transparency.	Hinders trust, regulatory acceptance, and deployment in safety-critical infrastructure systems.	Explainable AI (XAI); physics-guided architectures; uncertainty-aware learning frameworks.
Model Generalization & Transferability	Degraded performance when applied to new structures, materials, or environmental regimes.	Limits the scalability and reuse of trained models across infrastructure assets.	Domain adaptation; meta-learning; foundation models for SHM trained on multi-infrastructure datasets.
System-Level Integration	Fragmented development of sensing, analytics, and decision-making modules; limited interoperability.	Prevents deployment of end-to-end SHM systems for real-world asset management.	Integrated SHM platforms; digital twins; cyber–physical systems combining sensing, ML, and decision support.
Real-Time Intelligence	Latency due to data transmission and centralized processing; computational demands of deep models.	Restricts real-time monitoring capability and rapid decision-making.	Lightweight ML models; edge AI; adaptive and online learning for continuous SHM.

## Data Availability

No new data were created or analyzed in this study.

## Supplementary Materials

The following supporting information can be downloaded at: https://www.mdpi.com/article/10.3390/s26092641/s1. Supplementary File: S1, S2, and S3.

## Abbreviations

The following abbreviations are used in this manuscript:

AI	Artificial Intelligence
ANN	Artificial Neural Network
BPNN	Backpropagation Neural Network
CNN	Convolutional Neural Network
DFOS	Distributed Fiber Optic Sensor/Distributed Fiber Optic Sensing
DTS	Distributed Temperature Sensing
FBG	Fiber Bragg Grating
FEM	Finite Element Method
FNN	Feedforward Neural Network
FOS	Fiber Optic Sensor
IoT	Internet of Things
ML	Machine Learning
NDT	Non-Destructive Testing
OFDR	Optical Frequency Domain Reflectometry
OTDR	Optical Time Domain Reflectometry
PRISMA	Preferred Reporting Items for Systematic Reviews and Meta-Analyses
RUL	Remaining Useful Life
SHM	Structural Health Monitoring
SVM	Support Vector Machine
TS	Topic Search (Web of Science field tag)
WoS	Web of Science
