# Supplementary material for: Fiber-Optic Sensor-Based Structural Health Monitoring with Machine Learning: A Task-Oriented and Cross-Domain Review
Source: Sensors (Basel). 2026 Apr 24;26(9):2641. doi: 10.3390/s26092641 (PMC13165801; doi:10.3390/s26092641)
Supplement: Supplementary file 1 [file sensors-26-02641-s001.zip › S2_Keywords theme.pdf]

Keywords theme

Core Keywords (Must-Have)

These directly represent the paper's central contribution.

- Structural Health Monitoring (SHM)
- Fiber Optic Sensors (FOS)
- Machine Learning
- Intelligent Structural Monitoring
- Data-Driven SHM

🧵 Fiber Optic Sensing–Specific Keywords

Use these to capture sensor-focused literature.

- Fiber Bragg Grating (FBG) sensors
- Distributed Fiber Optic Sensing (DFOS)
- Optical fiber sensing
- Quasi-distributed fiber optic sensors
- Brillouin scattering (BOTDA, BOTDR)
- Rayleigh scattering
- Strain and temperature sensing

🤖 Machine Learning & Data Analytics Keywords

Target ML-enabled SHM studies.

- Machine learning–based SHM
- Deep learning
- Supervised learning
- Unsupervised learning
- Feature extraction
- Pattern recognition
- Data-driven damage detection
- Physics-informed machine learning

🧠 SHM Task-Oriented Keywords

Align with task-based review structure.

- Damage detection
- Damage localization
- Damage severity assessment
- Structural condition assessment
- Environmental and operational variability compensation

- Prognostics and health management (PHM)

🏗️ Infrastructure Application Keywords

Useful for domain-specific filtering.

- Civil infrastructure monitoring
- Transportation infrastructure
- Energy infrastructure monitoring
- Pipeline structural health monitoring
- Marine and offshore structures
- Aerospace structural monitoring

📊 Systematic Review & Methodology Keywords

Important for review-focused retrieval.

- Systematic review
- PRISMA guidelines
- Sensor–machine learning integration
- Smart sensing systems
- Intelligent infrastructure
